# Circulating uPA as a potential prognostic biomarker for resectable esophageal squamous cell carcinoma

**DOI:** 10.1097/MD.0000000000014717

**Published:** 2019-03-01

**Authors:** Xiao He, Xiaoling Xu, Guanxia Zhu, Hong Ye

**Affiliations:** a Department of Radiotherapy, Lishui People's Hospital, Lishui; b Key Laboratory of Diagnosis and Treatment Technology for Thoracic Cancer, Zhejiang Cancer Research Institute, Zhejiang Cancer Hospital, Zhejiang Cancer Center, Hangzhou, People's Republic of China.

**Keywords:** cyto-keratin 19 (CK-19), cyto-keratin 20 (CK-20), esophageal squamous cell carcinoma (ESCC), matrix metallopeptidase 9 (MMP9), urokinase type plasminogen activator (uPA)

## Abstract

Previous research showed that the 4 genes of matrix metallopeptidase 9 (MMP9), cyto-keratin 20 (CK20), cyto-keratin 19 (CK19) and urokinase type plasminogen activator (uPA) are detectable in the peripheral blood. All the 4 genes are related to tumor invasion and metastasis. However, whether their expression is associated with clinicopathologic factors and the prognosis of patients with esophageal squamous cell carcinoma (ESCC) is still confused. Expression levels of MMP9, CK20, CK19, and uPA were evaluated by quantificational real-time polymerase chain reaction (qRT-PCR) in peripheral blood of 205 ESCC patients who received radical resection. The cut-off value was 1000 copy numbers. Their impacts on clinicopathologic factors and survival were investigated. The uPA expression positively correlated with gender (*P* *=* .046) and tumor size (*P* = .046). Meanwhile, CK19 expression positively correlated with tumor size (*P* = .029), vascular invasion (*P* = .024), and CK20 expression positively correlated with tumor size (*P* = .035) and degrees of differentiation (*P* = .032). Moreover, the overexpression of MMP9 has a correlation with postoperative radiotherapy (*P* = .041) and chemotherapy (*P* = .012). Among the 4 genes, only uPA is a prognostic indicator for disease-free survival and overall survival both in univariate analysis and multivariate analysis (*P* = .015). This study suggests that circulating uPA mRNA in peripheral blood can serve as a potential unfavorable prognosis biomarker in ESCC. Further perspective, multi-center and large-scale study is still needed.

## 1 Introduction

Esophageal cancer (EC) is one of the most common cancers in Asia and the eighth cause of cancer related-death.^[1]^ Esophageal squamous cell carcinoma (ESCC) is the majority pathological subtype of EC in China.^[1]^ Surgery is one of the main treatments for EC patients and the 5-year survival rate in operable ESCC patients is only from 20% to 36%.^[2]^ The most responsible reasons for the failure of treatment and the majority of cancer-related deaths are regional recurrence and distant metastases. Thus, it is important to detect sensitive and specific biomarkers to identify potential postoperative EC patients who are tend to have early invasion and metastasis.

As we know, fibrin degradation and vascular formation effect can result in degradation of extracellular matrix and basement membrane which are the critical processes of tumor invasion and metastasis. The expression of matrix metallopeptidase 9 (MMP9), a member of the MMP family, can directly degrade the extracellular matrix. Thus, it serves a crucial role in cell migration. It has been reported to be involved in esophageal carcinoma metastasis,^[3]^ and high level of MMP9 expression is associated with lymph node metastasis in esophageal carcinoma.^[4]^ Cytokeratin 19 (CK19) and cytokeratin 20 (CK20) are the principal structural elements of the cytoskeleton of epithelial cells. They expressed during the process of transformation of the tissue from normal to tumor tissue.^[5]^ Therefore, CK19 and CK20 can serve as epithelial tumors metastasis molecular markers.^[6]^ ESCC is originated from the squamous epithelium cell. Thus, CK19 and CK20 may be the potential prognostic biomarker for ESCC. Urokinasetype plasminogen activator (uPA), as a proteolytic factor, can activate a variety of fibrinolytic enzymes. It is not only as a predictive biomarker, but also as a new therapeutic target because it can promote tumorous infiltration and metastasis by degrading extracellular matrix and basement membrane.^[7]^

Although, many studies have proved that MMP9, CK20, CK19, and uPA expression which detected by IHC and RT-PCR in tissue could serve as potential prognosis biomarker for various tumor patients.^[8–12]^ However, tissue biopsy is invasive and associated with a higher risk of complications for the patient. Up to date, only a few small-size studies^[13–15]^ have investigated the correlation between the expression of MMP9, CK19, CK20, and uPA in peripheral blood and the risk of EC . Therefore, we conducted this large-scale study to further determine whether MMP9, CK20, CK19, and uPA, detected in peripheral blood, can be a useful biomarker for patients with ESCC.

## 2 Methods

### 2.1 Patients and blood samples

Two hundred five patients with resectable ESCC diagnosed and treated at the Department of Thoracic Surgery of Zhejiang Cancer Hospital in China between September 2009 and March 2012 were included in the retrospective database for analysis. All patients were pathologically diagnosed by surgical specimens. This study was approved by the medical ethics committee of Zhejiang Cancer Hospital.

The following criteria should be satisfied:

1. patients who didn’t receive any preoperative treatment (chemotherapy and/or radiotherapy);
2. all patients should have R0 esophagectomy;
3. records with sufficient information including the follow-up information for analysis.

The exclusion criteria were as follows:

1. patients who presented with other malignant disease within 5 years;
2. patients with a noncurative resection;
3. patients who died within 30 days after operation.

Preoperative EDTA anticoagulated whole blood samples (2–3 ml) were collected from all patients with ESCC before surgery. Immediately after collection, blood samples were stored at −80°C until further processing.

### 2.2 RNA extraction and PCR

Total RNA was extracted using the Trizol reagent (Invitrogen Biotech, Shanghai, China). All RNA preparation and processing steps were conducted under RNAse-free conditions. Total RNA was diluted into 10 ng/μl and dissolved in diethylpyrocaronate-treated water (DEPC water), then stored at –80°C until use. RNA concentration was accessed by a spectrophotometer and the purity and integrity were detected using A260/A280 ratio. Electrophoresis on denaturated 1% agarose gel was used to further confirm the quality and concentration of the RNA samples.

The quality of the total RNA was checked by ethidium-stained 2% agarose gels. The reverse transcription reaction was carried out with a PrimeScriptTM RT reagent Kit (TaKaRa, Dalian, China) in 10 μl solution containing 2 μl of RNA extract, 2 μl 5×PrimeScript Buffer, 0.5 μl PrimeScript RT Enzyme Mix I, 0.5 μl Oligo dT Primer, 0.5 μl Random 6 mers and 4.5 μl RNase Free dH_2_O. For the synthesis of cDNA, reaction mixtures were incubated at 37°C for 15 minute, at 85°C for 5 second, and then held at 4°C.

SYBR green-based real-time quantitative polymerase chain reaction assays were used for the gene expression analysis of MMP9, CK20, CK19, and uPA according to the manufacturer's protocol of the Doagnostic Kit for MMP9/CK-19/CK-20/UPA-mRNA Kit (BioPerfectus technologies, Taizhou, China). The amounts of mRNAs were calculated by a standard curve constructed with the use of 5 μl quantitative standard reference which provided in the Kit. Next, 20 μl reaction mixtures consisted of 2 μl of cDNA solution, 17.8 μl gene-specific reaction mixtures and 0.2 Taq DNA polymerase was used to perform quantitative PCR, which was run on a 7500 Real-time PCR system (Applied Biosystems). Quantiative PCR was conducted with following cycle parameters: enzyme activation at 95°C for 5 minute, 40 cycles at 95°C for 30 second and 60°C for 30 second.

## 3 Statistical analysis

The association between MMP9, CK20, CK19, and uPA expression level and clinicopathological factors, such as gender, age, differentiation, tumor size, venous/lymphatic invasion, lymph node metastasis, and postoperative treatment, was analyzed using the *x*^2^ test or Fisher exact test. The disease-free survival (DFS) and overall survival (OS) of the patients was calculated by the Kaplan Meier method, and the survival curves were tested by the log-rank method. Multivariate Cox regression analysis was performed to include the parameters that were found to be significant by the univariate analysis. All results were analyzed using SPSS 18.00 statistical software (SPSS 18.0 Inc., Chicago, IL). A *P* value < .05 was considered to be statistically significant.

## 4 Results

### 4.1 Patient characteristics

Relationship between clinicopathological factors and MMP9, CK20, CK19, and uPA expression in the peripheral blood were presented in Table 1. This study examined a total of 205 ESCC patients (182 men and 23 women) with a mean age of 61 years (range, 39–81). To evaluate the correlation between the MMP9, CK20, CK19, and uPA mRNA levels and the clinicopathological characteristics, patients were divided into 2 groups according to copy numbers. The cut-off levels for MMP9, CK20, CK19, and uPA were set at 1000 copies, which were defined according to product manual suggestions. As showed in Table 1, a statistically significant association was observed between MMP-9 expression and postoperative radiotherapy (*P* *=* .041) or chemotherapy (*P* *=* .012). CK-20 high expression was associated with poor differentiation (*P* *=* .032) and larger tumor size (*P* *=* .035). A correlation between CK-19 high expression and larger tumor size (*P* *=* .022) and venous/lymphatic invasion (*P* = .024) were observed.

**Table 1:**
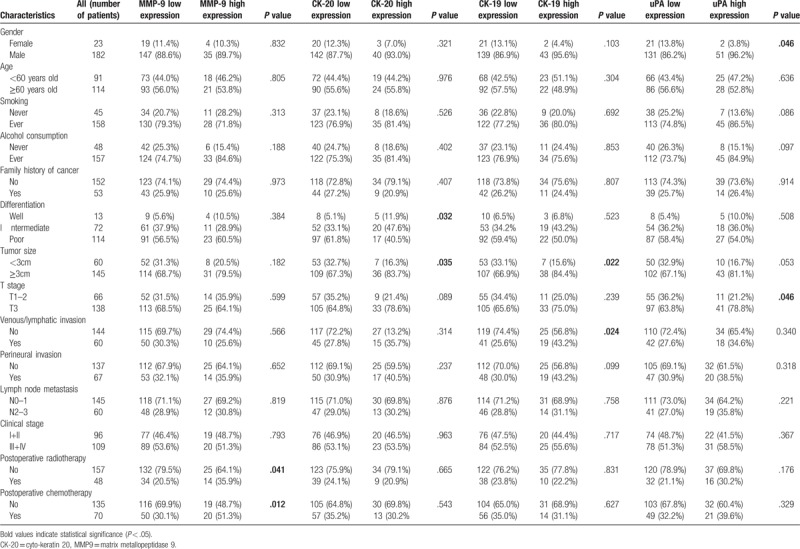
Expression levels of the 4 genes and associations with clinicopathological characteristics.

### 4.2 Univariate and multivariate Cox analysis of prognostic factors in OS and DFS

**Figure 1:**
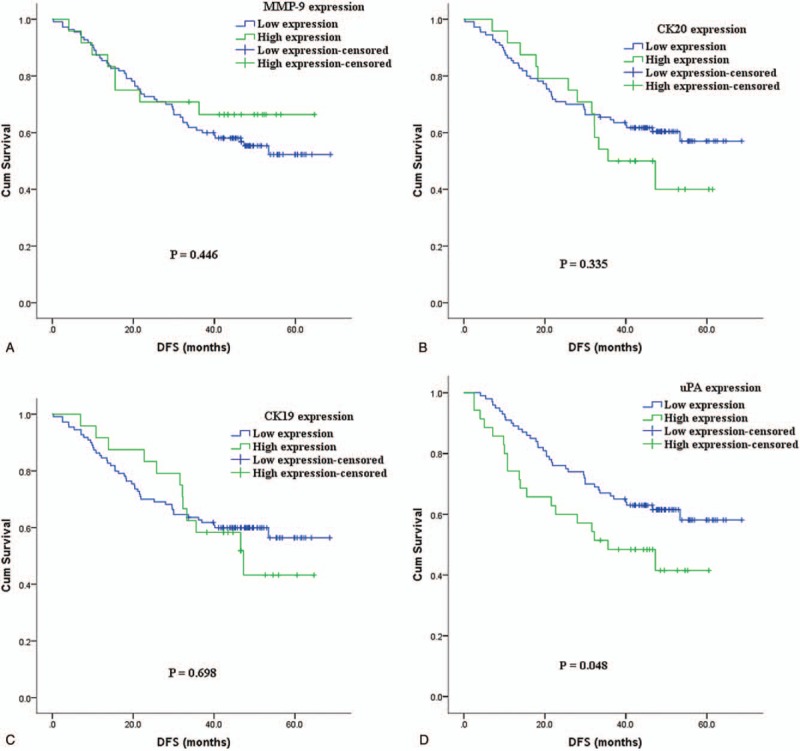
Kaplan–Meier disease-free survival estimates for patients with resectable esophageal squamous cell carcinoma according to circulating MMP9, CK20, CK19, and uPA expression levels. CK-19 = cyto-keratin 19, CK-20 = cyto-keratin 20, MMP9 = matrix metallopeptidase 9, uPA = urokinase type plasminogen activator.

**Figure 2:**
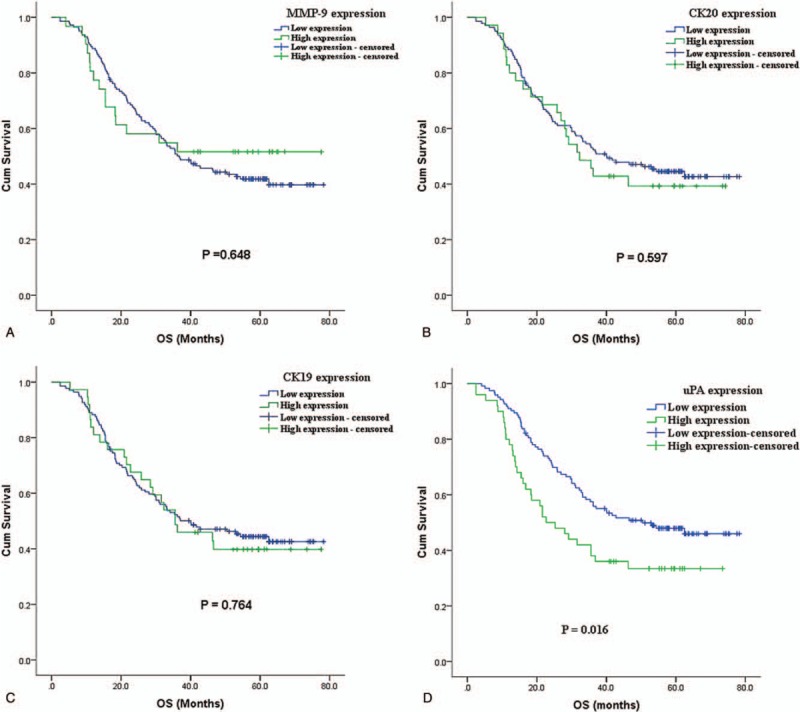
Kaplan–Meier overall survival estimates for patients with resectable esophageal squamous cell carcinoma according to circulating MMP9, CK20, CK19, and uPA expression levels. CK-19 = cyto-keratin 19, CK-20 = cyto-keratin 20, MMP9 = matrix metallopeptidase 9, uPA = urokinase type plasminogen activator.

Kaplan–Meier DFS and OS curves of the ESCC cancer patients according to the status of MMP9, CK20, CK19, and uPA levels were examined (Fig. 1). For DFS analysis, all 205 patients with underwent curative surgery were included for DFS analysis. The DFS of patients in the high uPA expression group showed significantly worse survival rates than those who were in the low uPA expression group (*P* = .048) (Fig. 1D). OS analysis revealed that the patients in the high uPA expression group had significantly worse survival rates than those who were in the low uPA expression group (*P* = .016) (Fig. 2D). These results suggest that high expression uPA is associated with poor prognosis in ESCC patients.

The results of univariate and multivariate Cox proportional hazard regression analysis for OS and DFS are shown in Tables 3. In the univariate analysis, tumor size, lymph node metastasis, T stage, and clinical stage showed significance for DFS and OS (Table 2). In the multivariate analysis, gender, smoking history, and T stage showed significance for both DFS and OS. These results suggest that uPA has independent prognostic value for both OS and DFS (Table 3).

**Table 2:**
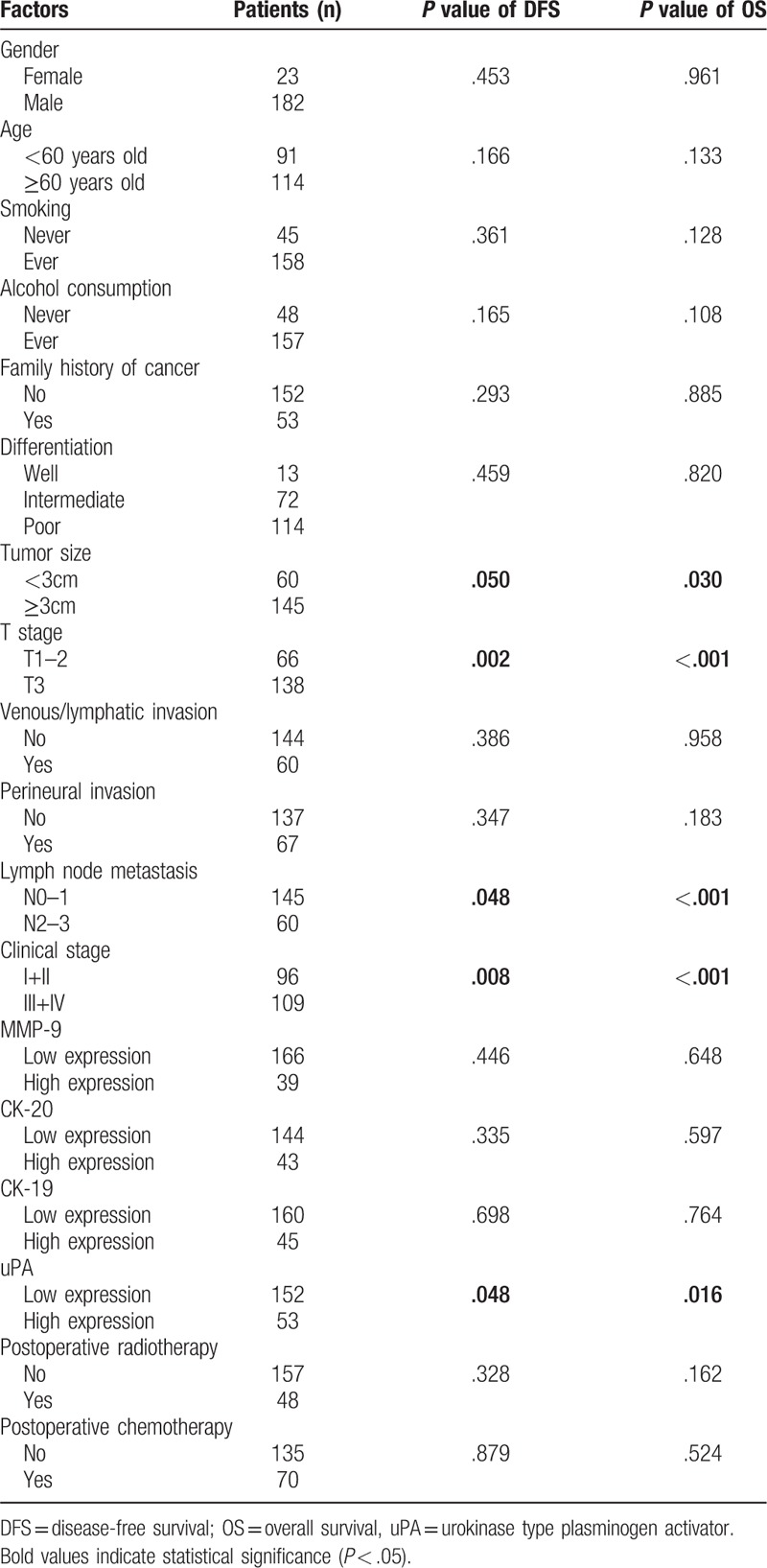
Univariate analysis of factors that influence the progression-free survival and overall survival.

**Table 3:**
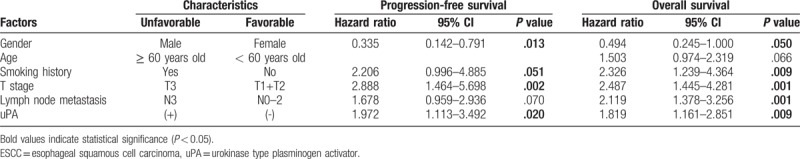
Multivariate analysis of progression-free survival and overall survival in ESCC.

## 5 Discussion

ESCC is an invasive malignant tumor with a high mortality rate (109.5 per 100,000).^[16]^ Obviously, the efficacy of simple surgical treatment is unsatisfactory.^[2]^ It is thus crucial to correctly identify those patients who have an increased risk of cancer recurrence and may benefit from adjuvant treatment. The aim of this study is to examine the expression level of 4 tumor-promoting biomarkers including MMP9, CK20, CK19, and uPA in the peripheral blood and the prognosis of patients with ESCC. As a result, none of the expression level of MMP9, CK20, or CK19, except uPA, was related to the DFS or OS of resectable ESCC.

uPA, a serine protease with multiple function, acts as risk assessment and a possible treatment target in many cancers,^[10,17,18]^ such as breast cancer,^[9,19,20]^ pancreatic cancer,^[21,22]^ prostate cancer,^[23,24]^ and ovarian cancer.^[25–27]^ In particular, uPA can accelerate tumor metastasis and promote tumor angiogenesis by degrading extracellular matrix (ECM) and basement membranes, such as vimentin and fibronectin, involving in epithelial-mesenchymal transition (EMT).^[27,28]^ Moreover, international guidelines (AGO, St. Gallen, ASCO) recommend the use of uPA and plasminogen activator inhibitor-1 (PAI-1) expression to better assess potential clinical benefit from adjuvant systemic treatment of breast cancer.^[29–31]^

The role of uPA overexpression in EC was also investigated. uPA overexpression was in tissue which accessed by immunohistochemistry related to clinical stage, differentiation and lymph node metastasis.^[32]^ However, we found no correlation between uPA overexpression and lymph node metastasis. Torzewski et al^[33]^ have proved that the intensity of uPA expression detected by immunohistochemistry was an independent risk prognostic factor in 150 potentially curatively resected ESCC patients. In our study, circulating uPA mRNA was found to be an independent predictor of a poor outcome in univariate and multivariate analysis. In addition, uPA expression was related to T stage, and uPA expression of the group with T3 stage were significantly higher than those of the group with T1–2 stage (*P* = .046).

Because a blood sample is more easily to be obtained, circulating uPA mRNA overexpression maybe a clinically useful, non-invasive screening strategy for ESCC patients. Furthermore, we usually access circulating protein using immunofluorescence, mass spectrometry, protein microarray, or Elisa. Obviously, qPCR which use to test mRNA expression is more economically and widespread use in most hospital. To our best knowledge, this is the first and large-scale study to demonstrate that uPA mRNA overexpression in the peripheral blood can be used as a reliable surrogate method to predict poor survival of resectable ESCC. However, the optimal cut-off value of circulating uPA mRNA expression is needed to be further validated. In addition, no healthy control group was evaluated in this study.

Based on our findings, we clearly demonstrated that circulating uPA mRNA in peripheral blood may be a promising prognostic predictor in ESCC patients postoperatively. It might be a potential new and interesting way in screening and monitoring of ESCC patients.

## Author contributions

**Conceptualization:** Xiao He.

**Data curation:** Xiaoling Xu.

**Formal analysis:** Xiaoling Xu, Guanxia Zhu.

**Investigation:** Xiao He.

**Project administration:** Guanxia Zhu.

**Validation:** Hong Ye.

**Writing – original draft:** Xiao He.

**Writing – review & editing:** Hong Ye.

## References

[1] Zheng S Vuitton L Sheyhidin I Northwestern China: a place to learn more on oesophageal cancer. Part one: behavioural and environmental risk factors. Eur J Gastroenterol Hepatol 2010;22:917–25. 20520561 10.1097/MEG.0b013e3283313d8b

[2] Izbicki JR Hosch SB Pichlmeier U Prognostic value of immunohistochemically identifiable tumor cells in lymph nodes of patients with completely resected esophageal cancer. N Engl J Med 1997;337:1188–94. 9337377 10.1056/NEJM199710233371702

[3] Xia T Tong S Fan K XBP1 induces MMP-9 expression to promote proliferation and invasion in human esophageal squamous cell carcinoma. Am J Cancer Res 2016;6:2031–40. 27725908 PMC5043112

[4] Yamamoto H Vinitketkumnuen A Adachi Y Association of matrilysin-2 (MMP-26) expression with tumor progression and activation of MMP-9 in esophageal squamous cell carcinoma. Carcinogenesis 2004;25:2353–60. 15333466 10.1093/carcin/bgh270

[5] Broers JL Ramaekers FC Rot MK Cytokeratins in different types of human lung cancer as monitored by chain-specific monoclonal antibodies. Cancer Res 1988;48:3221–9. 2452687

[6] Lam KY Loke SL Shen XC Cytokeratin expression in non-neoplastic oesophageal epithelium and squamous cell carcinoma of the oesophagus. Virchows Arch 1995;426:345–9. 7541275 10.1007/BF00191342

[7] Carriero MV Stoppelli MP The urokinase-type plasminogen activator and the generation of inhibitors of urokinase activity and signaling. Curr Pharm Des 2011;17:1944–61. 21711235 10.2174/138161211796718143

[8] Zeng R Duan L Kong Y Clinicopathological and prognostic role of MMP-9 in esophageal squamous cell carcinoma: a meta-analysis. Chin J Cancer Res 2013;25:637–45. 24385690 10.3978/j.issn.1000-9604.2013.11.03 PMC3872548

[9] Kolben T Augustin D Armbrust R Impact of guideline-based use of uPA/PAI-1 on patient outcome in intermediate-risk early breast cancer. Breast Cancer Res Treat 2016;155:109–15. 26643086 10.1007/s10549-015-3653-3

[10] Su SC Lin CW Yang WE The urokinase-type plasminogen activator (uPA) system as a biomarker and therapeutic target in human malignancies. Expert Opin Ther Targets 2016;20:551–66. 26667094 10.1517/14728222.2016.1113260

[11] Cao X Zhang L Feng GR Preoperative Cyfra21-1 and SCC-Ag serum titers predict survival in patients with stage II esophageal squamous cell carcinoma. J Transl Med 2012;10:197. 22999061 10.1186/1479-5876-10-197 PMC3548759

[12] Driessen A Nafteux P Lerut T Identical cytokeratin expression pattern CK7+/CK20- in esophageal and cardiac cancer: etiopathological and clinical implications. Mod Pathol 2004;17:49–55. 14631371 10.1038/modpathol.3800011

[13] Zhao W Wang X Qu B Clinical significance of plasma tissue factor pathway and urokinase-type plasminogen activator system in cancer patients. Chin Med J (Engl) 2002;115:702–4. 12133538

[14] Sasajima K Futami R Matsutani T Increases in soluble tumor necrosis factor receptors coincide with increases in interleukin-6 and proteinases after major surgery. Hepatogastroenterology 2009;56:1377–81. 19950795

[15] Mroczko B Kozlowski M Groblewska M The diagnostic value of the measurement of matrix metalloproteinase 9 (MMP-9), squamous cell cancer antigen (SCC) and carcinoembryonic antigen (CEA) in the sera of esophageal cancer patients. Clin Chim Acta 2008;389:61–6. 18155162 10.1016/j.cca.2007.11.023

[16] Chen W Zheng R Baade PD Cancer statistics in China, 2015. CA Cancer J Clin 2016;66:115–32. 26808342 10.3322/caac.21338

[17] Luporsi E Bellocq JP Barriere J uPA/PAI-1, Oncotype DX, MammaPrint ((R)). Prognosis and predictive values for clinical utility in breast cancer management. Bull Cancer 2015;102:719–29. 26235416 10.1016/j.bulcan.2015.05.003

[18] Zhang W Ling D Tan J Expression of urokinase plasminogen activator and plasminogen activator inhibitor type-1 in ovarian cancer and its clinical significance. Oncol Rep 2013;29:637–45. 23174953 10.3892/or.2012.2148

[19] Barajas-Castaneda LM Cortes-Gutierrez E Garcia-Rodriguez FM Overexpression of MMP-3 and uPA with diminished PAI-1 related to metastasis in ductal breast cancer patients attending a public hospital in Mexico City. J Immunol Res 2016;2016:8519648. 27975070 10.1155/2016/8519648 PMC5126427

[20] Lampelj M Arko D Cas-Sikosek N Urokinase plasminogen activator (uPA) and plasminogen activator inhibitor type-1 (PAI-1) in breast cancer - correlation with traditional prognostic factors. Radiol Oncol 2015;49:357–64. 26834522 10.2478/raon-2014-0049 PMC4722926

[21] Rybarczyk P Vanlaeys A Brassart B The transient receptor potential melastatin 7 channel regulates pancreatic cancer cell invasion through the Hsp90alpha/uPA/MMP2 pathway. Neoplasia 2017;19:288–300. 28284058 10.1016/j.neo.2017.01.004 PMC5345960

[22] Li J Kong F Wu K Sun W. miR-193b directly targets STMN1 and uPA genes and suppresses tumor growth and metastasis in pancreatic cancer. Mol Med Rep 2014;10:2613–20. 25215905 10.3892/mmr.2014.2558

[23] Serafin AM Akudugu JM Bohm L Influence of freeze-drying on the recovery of the tumour invasion markers uPA and PAI-1 from prostate cancer resections. Ann Clin Biochem 2015;52 (Pt 3):387–94. 25355777 10.1177/0004563214559546

[24] Sasaki H Klotz LH Sugar LM A combination of desmopressin and docetaxel inhibit cell proliferation and invasion mediated by urokinase-type plasminogen activator (uPA) in human prostate cancer cells. Biochem Biophys Res Commun 2015;464:848–54. 26182875 10.1016/j.bbrc.2015.07.050

[25] Cai S Zhang P Dong S Downregulation of SPINK13 promotes metastasis by regulating uPA in ovarian cancer cells. Cell Physiol Biochem 2018;45:1061–71. 29439245 10.1159/000487348

[26] Ghasemi A Hashemy SI Aghaei M RhoA/ROCK pathway mediates leptin-induced uPA expression to promote cell invasion in ovarian cancer cells. Cell Signal 2017;32:104–14. 28104444 10.1016/j.cellsig.2017.01.020

[27] Tang J Wang J Fan L cRGD inhibits vasculogenic mimicry formation by down-regulating uPA expression and reducing EMT in ovarian cancer. Oncotarget 2016;7:24050–62. 26992227 10.18632/oncotarget.8079 PMC5029683

[28] Moirangthem A Bondhopadhyay B Mukherjee M Simultaneous knockdown of uPA and MMP9 can reduce breast cancer progression by increasing cell-cell adhesion and modulating EMT genes. Sci Rep 2016;6:21903. 26906973 10.1038/srep21903 PMC4764826

[29] Goldhirsch A Wood WC Coates AS Strategies for subtypes--dealing with the diversity of breast cancer: highlights of the St. Gallen International Expert Consensus on the primary therapy of early breast cancer 2011. Ann Oncol 2011;22:1736–47. 21709140 10.1093/annonc/mdr304 PMC3144634

[30] Hanf V Schutz F Liedtke C AGO recommendations for the diagnosis and treatment of patients with advanced and metastatic breast cancer: update 2014. Breast Care (Basel) 2014;9:202–9. 25177262 10.1159/000363551 PMC4132248

[31] Harris L Fritsche H Mennel R American society of clinical oncology 2007 update of recommendations for the use of tumor markers in breast cancer. J Clin Oncol 2007;25:5287–312. 17954709 10.1200/JCO.2007.14.2364

[32] Jiang JT Zhang LF Zhou B Relationships of uPA and VEGF expression in esophageal cancer and microvascular density with tumorous invasion and metastasis. Asian Pac J Cancer Prev 2012;13:3379–83. 22994764 10.7314/apjcp.2012.13.7.3379

[33] Torzewski M Sarbia M Verreet P Prognostic significance of urokinase-type plasminogen activator expression in squamous cell carcinomas of the esophagus. Clin Cancer Res 1997;3 (12 Pt 1):2263–8. 9815623

